# *SlbZIP38*, a Tomato bZIP Family Gene Downregulated by Abscisic Acid, Is a Negative Regulator of Drought and Salt Stress Tolerance

**DOI:** 10.3390/genes8120402

**Published:** 2017-12-20

**Authors:** Yanglu Pan, Xin Hu, Chunyan Li, Xing Xu, Chenggang Su, Jinhua Li, Hongyuan Song, Xingguo Zhang, Yu Pan

**Affiliations:** 1 Key Laboratory of Horticulture Science for Southern Mountainous Regions, Ministry of Education, Southwest University, Chongqing 400715, China; pyl77111@163.com (Y.P.); emezhx@126.com (X.H.); 18996409742@163.com (C.L.); xuxg1016@163.com (X.X.); suchenggang@swu.edu.cn (C.S.); ljh502@swu.edu.cn (J.L.); yuahs@sohu.com (H.S.); 2 College of Horticulture and Landscape Architecture, Southwest University, Chongqing 400715, China

**Keywords:** *Solanum lycopersicum*, ABA, bZIP transcription factor, drought stress, salt stress, *SlbZIP38*

## Abstract

The basic leucine zipper (bZIP) transcription factors have crucial roles in plant stress responses. In this study, the bZIP family gene *SlbZIP38* (GenBank accession No: XM004239373) was isolated from a tomato (*Solanum lycopersicum* cv. Ailsa Craig) mature leaf cDNA library. The DNA sequence of *SlbZIP38* encodes a protein of 484 amino acids, including a highly conserved bZIP DNA-binding domain in the C-terminal region. We found that *SlbZIP38* was differentially expressed in various organs of the tomato plant and was downregulated by drought, salt stress, and abscisic acid (ABA). However, overexpression of *SlbZIP38* significantly decreased drought and salt stress tolerance in tomatoes (Ailsa Craig). The findings that *SlbZIP38* overexpression reduced the chlorophyll and free proline content in leaves but increased the malondialdehyde content may explain the reduced drought and salt tolerance observed in these lines. These results suggest that *SlbZIP38* is a negative regulator of drought and salt resistance that acts by modulating ABA signaling.

## 1. Introduction

Plant growth and crop productivity are limited by various adverse environmental stresses, including drought, high-salinity, and low temperature, which were significantly reduced the crop productivity [1]. Extensive functional studies have demonstrated that the plant hormone abscisic acid (ABA) plays a role in the response to biotic and abiotic stress [2,3,4]. The mechanisms through which this hormone works have become increasingly clear since the core signaling pathways of ABA have been identified [5,6,7]. In response to drought, salt, and cold stress, ABA triggers the induction of dehydration resistance genes [8], including the late embryogenesis abundant (LEA) genes and those encoding transcription factors, water transporters such as aquaporin, catalase, protein kinases, and phosphatases [9]. The expression of these genes protects cells and enables the plant to adapt to the stressful environment [10,11].

The basic leucine zipper (bZIP) family of transcription factors is one of the largest transcription factor (TF) families in plants. To date, 75 bZIP genes have been identified in *Arabidopsis thaliana* [12], 89 in *Oryza sativa* (rice) [13], 125 in *Zea mays* (maize) [14], and 131 in *Glycine max* (soybean) [15]. Members of this family are classified into different subgroups based on the presence of the leucine zipper dimerization motif and the specific DNA-binding domain including the DELAY OF GERMINATION 1 (DOG1) and Multifunctional Mosaic Region (MFMR) conserved areas [13,16]. These transcription factors respond to different abiotic stresses by recognizing and binding to genetic elements containing an ACGT core motif, such as a G box containing a CACGTG core motif, a C box containing a GACGTC core motif, an A box containing a TACGTA core motif, an ABA-responsive element (ABRE) containing a CCACGTGG core motif [17], and an as-1 (activation sequence = 1) core motif [18]. Thus, a series of bZIP genes have been shown to function in the ABA-regulated response to various abiotic stresses, including drought and salt [19,20]. In *Arabidopsis*, ABF3 [21] and AREB2/ABF4 [22] are involved in ABA regulation, and the overexpression of these genes improves drought tolerance [19]. In addition, transcription factors AREB1, AREB2, and ABF3 involved in multiple stress responses and require ABA for full activation, while AREB1/ABF2 is involved in posttranslational regulation [23,24,25]. *AtbZIP2*, *AtbZIP11*, and *AtbZIP53* function in developmental processes and stress responses, particularly the response to salt stress [26,27], and *AtbZIP1*, *AtbZIP11*, *AtbZIP24*, and *AtbZIP44* function in response to low temperature stress [26,28]. In rice, *OsbZIP16* [29], *OsbZIP23* [30,31,32], *OsbZIP33* [33], *OsbZIP45* [32], *OsbZIP46* [34], *OsbZIP52* [35], *OsbZIP71* [36], and *OsbZIP72* [37] are involved in the drought stress response; *OsbZIP23* and *OsbZIP71* have roles in salt stress tolerance [31,36]; and *OsbZIP52* and *OsbZIP71* function in the cold stress response [35,36]. Intriguingly, the native *OsbZIP46* gene, which encodes a constitutively active form of OsbZIP46 (OsbZIP46CA1), increases ABA sensitivity with no positive effect on drought resistance. By contrast, a protein kinase (SAPK6) involved in ABA signaling has been shown significantly enhance drought resistance [34,38]. In *Triticum aestivum* (wheat), most known bZIPs, i.e., TaABP1 [39], TabZIP60 [40], TaAREB3 [41], and WABI5 [42], are primarily involved in the drought stress response, but also have a role in attenuating salt and cold stress. By contrast, TabZIP14B is only involved in salt stress and cold stress [43]. *ABP9*, a maize bZIP transcription factor, plays a role in tolerance to drought, salt, and freezing temperatures through ABA signaling and by controlling the accumulation of reactive oxygen species [44]. Maize bZIP60 and bZIP72 increase drought and salt tolerance when heterologously expressed in *Arabidopsis* [1,45]. Similarly, *GmbZIP1*, *GmbZIP110*, and *GmFDL19* enhance salt stress tolerance in soybean [46,47,48]. A few bZIPs function in abiotic stress responses in Solanaceae, namely SlAREB1 [49,50], LebZIP2 [51], CabZIP1 [27], and ABZ1 [52]. SlAREB1 was identified in salt- and drought-resistant wild and cultivated tomatoes [50]. LebZIP2 is induced by salt and drought stress and may participate in an abiotic stress signaling pathway by regulating the expression of *NbNOA1* or *NbNR* in *Nicotiana benthamiana* [51]. In *Capsicum annuum*, CabZIP1 enhances disease resistance and environmental stress tolerance [27]. ABZ1 proteins are involved in the negative regulation of gene expression under anaerobic conditions [52].

In tomatoes (*Solanum lycopersicum*), which are both major crops and model systems for fruit development, and a total of 69 members in the *SlbZIP* family have been characterized to date. Some of these members have been associated with various abiotic and biotic stresses as well as the response to light [16]. In this study, we isolated a gene encoding a bZIP transcription factor, *SlbZIP38*, from tomatoes, analyzed its expression profiles under different abiotic stress conditions and hormone treatments, and characterized its role in stress tolerance. We found that overexpression of *SlbZIP38* substantially reduced drought tolerance of tomatoes (*Solanum lycopersicum* cv. Ailsa Craig).

## 2. Materials and Methods 

### 2.1. Plant Materials and Growth Conditions

The seeds of *Solanum lycopersicum* cv. Ailsa Craig used in this study were grown at Southwest University, Chongqing, China. They were surface-sterilized with 70% ethanol for 3 min and 1% NaClO for 10 min, followed by several rinses with ddH_2_O, and then sown in the 15 cm diameter pots containing a soil: Perlite mixture (1:1) and grown under standard greenhouse conditions (14 h daylight and 10 h darkness at 25 ± 2 °C). Plants were watered every 3 days.

The Wild type (WT) and *SlbZIP38* transgenic seedlings (T_2_, generation) were pre-grown as described above. One-month-old seedlings were used to perform the stress treatments. The phenotype of WT and *SlbZIP38* transgenic tomatoes was observed after 7 days.

### 2.2. Sequence and Phylogenetic Analysis

Both Basic Local Alignment Search Tool (Bethesda, MD, USA) and hidden Markov model profile searches were obtained from the tomato genome database at the Sol Genomics Network [53]. The sequences were used as queries for searches of the National Center of Biotechnology Information Conserved Domain Database search databases [54] to verify the presence of the bZIP domain. Multiple sequence alignments of the full SlbZIP protein, basic regions, and leucine zipper domains were performed using COBALT of the National Center of Biotechnology Information (Bethesda, MD, USA). Phylogenetic trees were constructed using Molecular Evolutionary Genetics Analysis (MEGA) 6.0 [55], and the neighbor-joining (NJ) method with the default parameters manually adjusted, using the JTT+I+G substitution model and 1000 bootstrap replicates [55].

### 2.3. Abiotic Treatment Assays

The various abiotic stress treatments were performed as our described formal rules [56]. Firstly, one-month-old seedlings of WT or transgenic plants were transferred to 9-cm pots for further analysis. Then, they were treated with various adverse environmental stresses, including low and high temperatures (4 °C, 22 °C and 37 °C for 4 h, 8 h, and 24 h); flooding (for 2, 4, and 8 days); wilt and recovery (water was withheld for one week and then plants were rewatered until recovery); and salt stress (0, 100, 200, 300, 400 and 500 mM NaCl treatment for 24 h). In addition, the seedlings were treated with abscisic acid (ABA, 100 μM), gibberellic acid (GA, 100 μM), salicylic acid (SA, 100 μM), jasmonic acid (JA, 100 μM), and ethylene (Eth, 1 mM). The light cycle was 24 h calculated from the first day at 8:00 a.m. to the next 8:00 a.m. Finally, all harvested samples were immediately immersed in liquid nitrogen and stored at −80 °C until analysis. In each case, individual plants were used for each treatment with three biological replicates.

### 2.4. RNA Isolation and Quantitative Real-Time PCR Analysis

Total RNA from the leaves, stems, and roots of tomatoes, isolated using RNAiso Plus (TaKaRa, Dalian, China) as described in the manufacturer’s instructions, were subjected to DNase I (TaKaRa, Dalian, China) treatment. The DNase-treated RNA was reverse-transcribed using a PrimeScript^TM^ RT Reagent Kit (TaKaRa, Dalian, China). Quantitative real-time PCR (qRT-PCR) was performed on a CFX96 Real-Time PCR system (Bio-Rad Laboratories, Hercules, CA, USA) with Eva Green SMA (Bio-Rad Laboratories, Hercules, CA, USA). The *SlELF-α* gene (GenBank accession number: X14449) was used as an internal control for normalization [57]. Relative expression of the detected gene was calculated using the 2^−ΔΔCt^ method. The primer sequences for the qRT-PCR assays are as follows: *ELF-α* and *SlbZIP38-*Q (Table 1). The expression of key genes in the ABA signaling pathway [49,58,59,60,61,62] was analyzed by RT-qPCR using the primers shown in Table 1.

### 2.5. Chlorophyll Content Measurement

For chlorophyll measurement, six individual leaf samples were removed from plants with distinct phenotypes, weighed, and extracted with 10 mL 80% aqueous acetone (*v*/*v*). The extract was centrifuged at 4000× *g* for 5 min in the room temperature and the absorbance was recorded at 663 and 646 nm using a Lambda 900 scanning spectrophotometer (PerkinElmer, Waltham, MA, USA). Total chlorophyll content was calculated according to the formula: Total Chlorophyll (µg mL^−1^) = 20.29 A_646_ + 8.02 A_663_ [63].

### 2.6. SlbZIP38 Overexpression Vector Construction and Tomato Transformation

The full-length cDNA of *SlbZIP38* was amplified by polymerase chain reaction (PCR) with the SlbZIP38 primer (Table 1) from a tomato (*Solanum lycopersicum* cv. Ailsa Craig) mature leaf cDNA library. The primers of *SlbZIP38* was designed by primer premier 5 according to the cDNA sequence (GenBank accession No: XM004239373). The PCR fragments were cloned into pESAY-Blunt, then digested by *BamHI* and *Ecl136II* and subcloned into the binary expression vector PVCT2024, which was also digested with *BamHI* and *Ecl136II*. The constructs were transformed into the *Solanum lycopersicum* cv. Ailsa Craig plants by *Agrobacterium tumefaciens* strain LBA4404 [64].

### 2.7. Measurement of Proline Content

Proline content was measured following a previously reported method with some modification [63]. Briefly, 0.5 g of leaf tissue was treated with 3% (*w*/*v*) aqueous sulphosalicylic acid for 10 min, and then the supernatants were collected by pipette following centrifugation at 500× *g* for 15 min in the room temperature, which were pipetted and subsequently treated with acid-ninhydrin at 90 °C for 1 h. Then, the reaction was terminated in an ice bath and the colored complex extracted in toluene. The absorbance was read at 520 nm. Proline concentration was measured using a calibration curve and expressed as μg proline g^−1^ fresh weight. The standard curve for proline was prepared by dissolving proline in 3% sulfosalicylic acid to cover the concentration range 0.5–10 μg mL^−1^ [65,66].

### 2.8. Malondialdehyde Content Detection

The level of lipid peroxidation was determined in terms of malondialdehyde (MDA) content. Briefly, 0.2 g leaf sample was homogenized in 4 mL of 1.0% trichloroacetic acid (TCA) and centrifuged at 15,000× *g* for 5 min at room temperature. Then, 4 mL of 20% TCA containing 0.5% thiobarbituric acid (TBA) was added to 1 mL supernatant. The mixture was blended and incubated at 95 °C in water for 30 min, and the reaction was terminated in an ice bath for 10 min. The mixture was centrifuged at 10,000× *g* for 10 min at room temperature. The absorbance of the supernatant was recorded at 532 and 600 nm. The MDA equivalent was calculated as follows: MDA (nmol/mL fresh weight) = ((A_532_ − A_600_)/155,000) × 10^6^ [67].

### 2.9. Statistical Analysis

The mean values of qRT-PCR, MDA, proline and chlorophyll were taken from the measurements of three independent biological replicates and ‘Standard Error’ of the means was calculated. Data represent the means ± Standard error(SE, *n =* 3)—significantly different values from the WT at the *p* < 0.05 and *p* < 0.01 level, respectively, as determined by Student’s *t*-test, the expression profiles of *SlbZIP38* under abiotic stress treatments, and the untreated samples at the same time point were used as the reference control. For the analysis of SlbZIP38-overexpressing plants under drought and salt stress, the WT was used as a reference control for Student’s *t*-test statistical analysis.

## 3. Results

### 3.1. Structural and Phylogenetic Analysis of SlbZIP38 in Solanum lycopersicum

**Figure 1:**
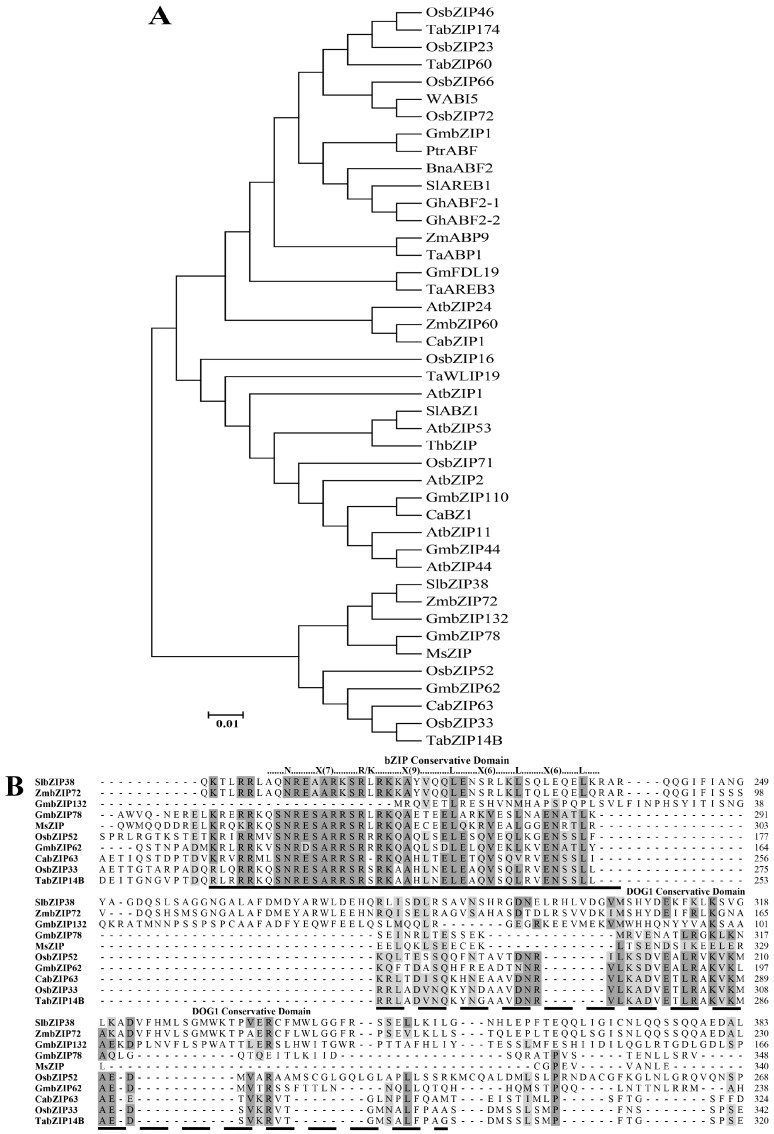
Characterization of SlbZIP38. (**A**) the amino acid sequences of SlbZIP38 and of 42 bZIP transcription factors from different plant species were aligned using ClustalW2. The phylogenetic relationship was constructed using MEGA6 software (Version 6.06.) with the neighbor-joining tree method with 1000 bootstrap replicates. The scale bar indicates 0.01 amino acid substitutions per site; (**B**) comparison of conserved bZIP domains of SlbZIP38. Sequences were aligned using COBALT of the National Center of Biotechnology Information (Bethesda, MD, USA). Conserved amino acids are highlighted in light gray (identity rate > 65%) and dark gray (identity rate > 85%); At (*Arabidopsis thaliana*), Bna (*Brassica napus*), Ca (*Capsicum annuum*), Gh (*Gossypium hirsutum*), Gm (*Glycine max*), Ms (*Medicago sativa* Linn), Os (*Oryza sativa*), Ptr (*Poncirus trifoliata*), Sl (*Solanum lycopersicum*), Ta (*Triticum aestivum*), Th (*Tamarix hispida*), Zm (*Zea mays*).

The 1455-bp full-length cDNA sequence of the *SlbZIP38*, a basic leucine zipper (bZIP) transcription factor was obtained from a cDNA library prepared from mature tomatoes (*Solanum lycopersicum* cv. Ailsa Craig) leaves. The largest open reading frame encoded a protein of 484 amino acids and had a predicted molecular weight of 53.7 kDa with a pI of 6.58. We collected forty-two bZIP protein sequences linked to abiotic stress were collected from National Center of Biotechnology Information (NCBI) (Bethesda, MD, USA) for *Solanum lycopersicum* (tomato), *Capsicum annuum* (pepper), *Arabidopsis thaliana*, *Gossypium hirsutum* (cotton), *Zea mays* (maize), *Glycine max* (soybean), and *Triticum aestivum* (wheat) (Figure 1). Using these sequences, we constructed a phylogenetic tree and found that ZmbZIP72 [1], GmbZIP132 [68], GmbZIP78 [15], and MsbZIP [69] were clustered to the same branch as SlbZIP38 (Figure 1A). Amino acid alignments of these bZIP proteins revealed that SlbZIP38 shared high levels of amino acid sequence similarity with ZmbZIP72, GmbZIP132, GmbZIP78, and MsbZIP had a conserved bZIP DNA-binding domain spanning a 52-amino acid long basic region (N-x7-R/K-x9) and a DOG1 motif composed of 79 amino acid residues (Figure 1B), indicating that the phylogenetic analysis corresponds with the results of Multiple EM for Motif Elicitation (MEME) prediction.

### 3.2. The Expression Profiles of SlbZIP38 under Abiotic Stress Treatments

**Figure 2:**
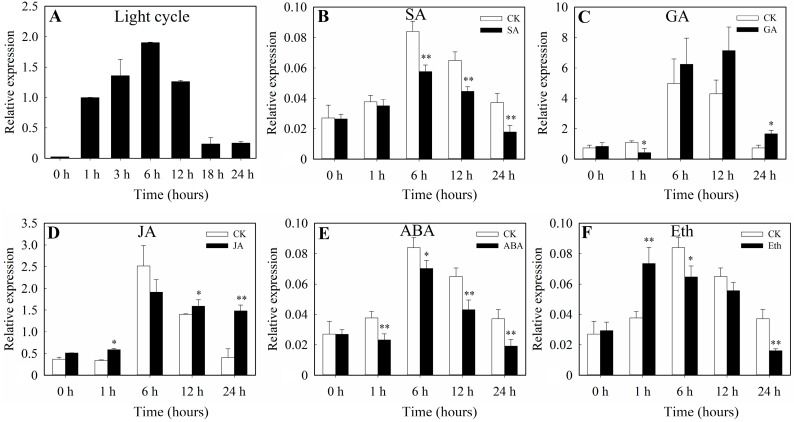
Expression profiles of *SlbZIP38* in the leaves of Ailsa Craig under exogenous hormone treatments. (**A**) light cycle treatment; (**B**) 100 μM salicylic acid (SA) treatment; (**C**) 100 μM gibberellic acid (GA) treatment; (**D**) 100 μM jasmonic acid (JA) treatment; (**E**) 100 μM exogenous abscisic acid (ABA) treatment; (**F**) 1 mM ethylene (Eth) treatment. CK, the Wild type (WT) plant treated with water which equal volume to hormone serving as the control. Gene expression analyses were examined by RT-qPCR and the expression values were calculated using the 2^−ΔΔCt^ method and normalized to expression of the *ELF-α* housekeeping gene. Data represent the means ± SE (*n =* 3). Significantly different values from CK at the * *p* < 0.05 and ** *p* < 0.01 level, respectively, as determined by Student’s *t*-test.

We then analyzed the expression pattern of *SlbZIP38* to shed light on the functional significance of the gene. Expression levels peaked 6 h after the introduction of abiotic stress or the start of phytohormone treatment and had returned to the control levels after 18 h. *SlbZIP38* expression was positively affected by the light cycle; its expression peaked after 6 h (Figure 2A). Real-time quantitative reverse transcription PCR (RT-qPCR) was used to detect the expression of *SlbZIP38* in leaves treated with the phytohormones gibberellic acid (GA), jasmonic acid (JA), salicylic acid (SA), abscisic acid (ABA), and ethylene (Eth). *SlbZIP38* was always expressed at lower levels in the treatment conditions compared to the control for the SA and ABA treatments (Figure 2B,E). By contrast, *SlbZIP38* expression showed different expression tendencies after treatment with GA, JA, and Eth (Figure 2C,D,F), resulting in higher expression compared to the control at some time points, but lower expression at others. These results indicate that *SlbZIP38* expression is negatively regulated by SA and ABA, and is also induced by light.

### 3.3. SlbZIP38 Expression Profiles under Various Abiotic Stresses

Using reverse transcription quantitative PCR (RT-qPCR), we examined *SlbZIP38* expression patterns in the roots, stems, and leaves of one-month-old tomato seedlings subjected to various abiotic stressors, as outlined below.

#### 3.3.1. Temperature Treatment

**Figure 3:**
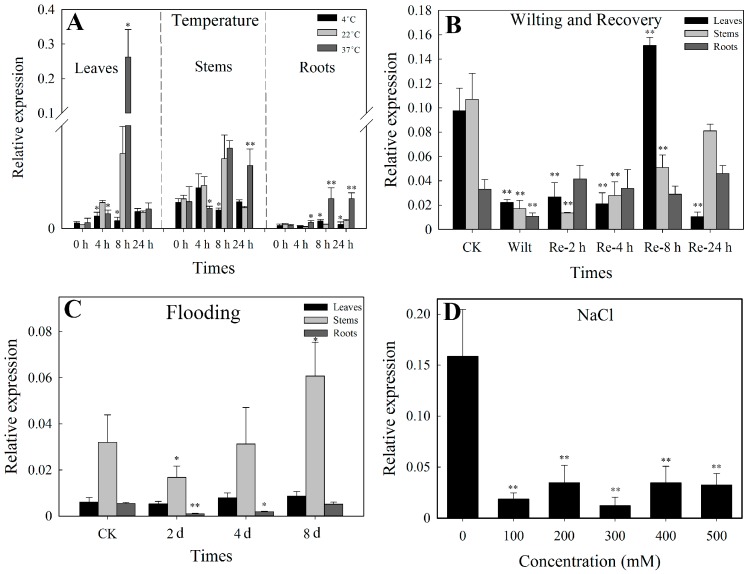
Quantitative Real-time-PCR (qRT-PCR) analysis of *SlbZIP38* expression under different abiotic stressors. *SlbZIP38* expression in Ailsa Craig tomato plants (**A**) with exposure to different temperatures; plants under 22 °C serving as the control (CK); (**B**) wilting and recovery from water stress; CK, samples from untreated plants serving as the control; (**C**) under flooding; CK, samples from untreated plants serving as the control; and (**D**) exposure to different concentration of NaCl; Plants under 0 mM NaCl serving as the control. Data represent the means ± SE (*n =* 3). Significantly different values from the control at the * *p* < 0.05 and ** *p* < 0.01 level, respectively, as determined by Student’s *t*-test.

Compared to the control (22 °C), we found that, for short durations of treatment (4 h), *SlbZIP38* expression was reduced in leaves after exposure to 4 °C and 37 °C, but it was significantly increased in roots at 37 °C. Expression peaked at 8 h of treatment at 37 °C in the leaves, stems and roots (Figure 3A). However, no significant changes in *SlbZIP38* expression were detected in leaves and stems after 24 h of treatment at 4 °C treatment (Figure 3A). These data indicate that expression of *SlbZIP38* expression was induced in roots by low (4 °C) and high (37 °C) temperatures. Furthermore, *SlbZIP38* expression is readily induced by the high (37 °C) temperature.

#### 3.3.2. Drought Stress

Compared with normally watered plants, *SlbZIP38* expression was significantly reduced in the leaves, stems, and roots of wilted plants that had not been watered for one week (Figure 3B, Student’s *t*-test, *p* < 0.01). Furthermore, *SlbZIP38* transcript was present at normal levels in the roots of these plants by 2 h after rewatering, but not in the leaves and stems (Figure 3B). By contrast, the levels of *SlbZIP38* transcript in the leaves peaked 8 h after rewatering, and then dramatically decreased to a minimum again after 24 h (Figure 3B). These data indicate that *SlbZIP38* is readily induced by drought.

#### 3.3.3. Flooding 

As shown in Figure 3C, *SlbZIP38* expression in leaves was not affected by flooding in the leaves, but was significantly reduced in the stems and roots after two days of flooding. After 8 d of flooding, *SlbZIP38* expression increased to the higher levels (2-fold that of the untreated control, CK) in the stems and to normal levels in the roots (Figure 3C). Our results showed that *SlbZIP38* expression was induced by water stress in the stems and roots, but not in the leaves. 

#### 3.3.4. NaCl Stress

To determine the plant’s response to high salt conditions, one-month-old tomato seedlings were placed on various concentrations of NaCl for 24 h. Compared with CK, the expression levels of *SlbZIP38* were markedly reduced by exposure to salt stress (Figure 3D, Student’s *t*-test, *p* < 0.01). Thus, *SlbZIP38* expression is associated with salt treatment.

### 3.4. Performance Analysis of SlbZIP38-Overexpressing Plants under Drought Stress

**Figure 4:**
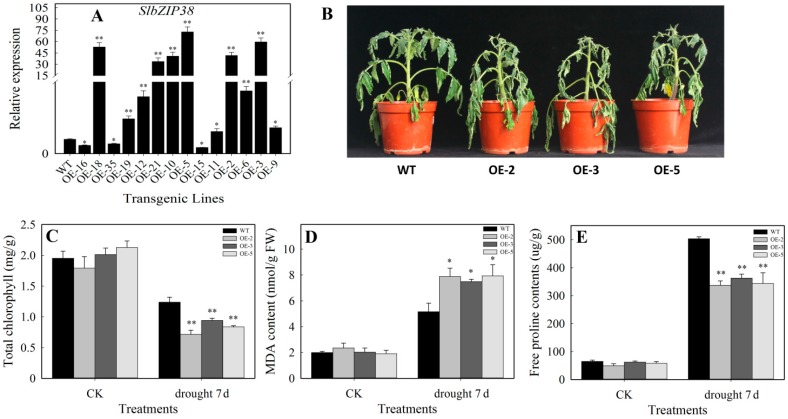
Drought tolerance of *SlbZIP38*-overexpressing and Wild type (WT) plants. (**A**) *SlbZIP38* expression in Wild type (WT) and transgenic plants (OE-2, OE-3, OE-5), as determined by qRT-PCR; (**B**) the performance of *SlbZIP38*-overexpressing and WT plants under drought stress. One-month-old transgenic (T2 generation) and WT plants were not watered for seven days (**C**–**E**); comparisons of chlorophll (**C**), malondialdehyde (MDA) (**D**), and free proline (**E**) content in the transgenic and WT plants after 7 d of dehydration. CK, samples from untreated plants serving as the control. Data represent the means ± SE (*n =* 3). Significantly different values from WT at * *p* < 0.05 and ** *p* < 0.01 level, respectively, as determined by Student’s *t*-test.

We then transgenically overexpressed *SlbZIP38* driven by the 35S promoter in tomato plants. Fourteen independent *SlbZIP38* transgenic lines were obtained. In 11 of these, *SlbZIP38* expression was higher than that in the wild type (WT), but the expression level of three lines (OE-15, OE-16 and OE-35, OE: Overexpression) was significantly lower (Figure 4A). For further analysis, we selected OE-2, OE-3, and OE-5, in these lines, *SlbZIP38* expression was approximately 41.5-, 59.5-, and 72.7-fold that of the WT, respectively (Figure 4A). 

To examine whether *SlbZIP38* overexpression conferred resistance to drought stress, one-month-old OE-2, OE-3, OE-5, and WT plants were not watered for seven days. Before stress treatment, no evident phenotypic differences were identified between the transgenic and WT plants. After the drought stress treatment, the transgenic plants showed more severe wilting and shrinking of the leaves than the WT (Figure 4B). Thus, the transgenic plants were more susceptible to drought stress than the WT plants were. The levels of chlorophyll, malondialdehyde (MDA) and free proline are used as an indicator of tolerance to abiotic stresses, such as drought, salinity, and extreme temperatures [49,70,71,72,73]. To evaluate the effects of drought stress on the performance of *SlbZIP38*-overexpressing transgenic plants, we thus compared the levels of chlorophyll, malondialdehyde (MDA), and free proline in the leaves of OE-2, OE-3, and OE-5 plants under drought stress with those in the WT. Following drought stress, the total chlorophyll content was significantly decreased in all plants examined (Student’s *t*-test, *p* < 0.01); this change was more pronounced in the transgenic plants than in the WT (Figure 4C). Before stress, the physiological levels of MDA and free proline were similar between transgenic and WT plants, whereas they were dramatically increased (Student’s *t*-test, *p* < 0.05) in the transgenic plants following drought treatment (Figure 4D). The free proline content of *SlbZIP38*-overexpressing plants was significantly lower (Student’s *t*-test, *p* < 0.01) than that of WT plants (Figure 4E). These results suggest that overexpression of the native *SlbZIP38* gene may has a negative effect on drought stress tolerance.

### 3.5. Performance Analysis of SlbZIP38-Overexpressing Plants under Salt Stress

**Figure 5:**
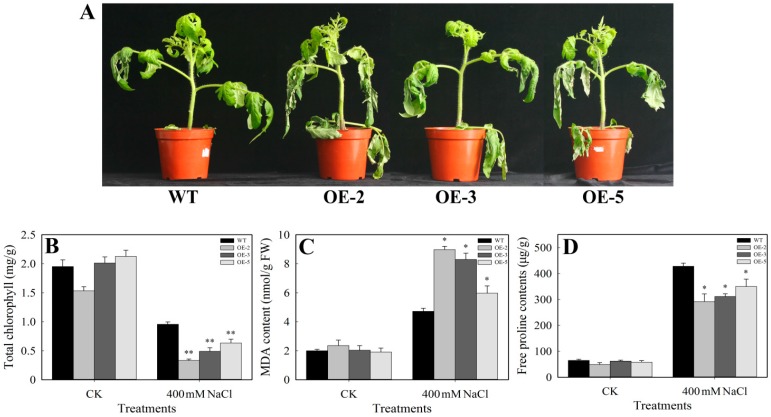
Salt tolerance of *SlbZIP38*-overexpressing and WT plants. (**A**) the performance of *SlbZIP38*-overexpressing and WT plants under salt stress. One-month-old transgenic (T2 generation) and WT plants were treated with 400 mM NaCl for five days; (**B**–**D**) comparisons of chlorophll (**B**), malondialdehyde (MDA) (**C**), and free proline (**D**) content of transgenic and WT plants. CK, samples from untreated plants serving as the control. Data represent the means ± SE (*n =* 3). Significantly different values from WT at * *p* < 0.05 and ** *p* < 0.01 level, respectively, as determined by Student’s *t*-test.

Homozygous transgenic plants OE-2, OE-3, and OE-5 of the T_2_ generation were subjected to salt tolerance testing with 400 mM NaCl. Whereas the transgenic plants displayed chlorosis and leaf fall after treatment with 400 mM NaCl, the WT plants were more resistant to salt stress (Figure 5A). We also measured the levels of chlorophyll, MDA, and free proline in plants subjected to salt stress. In contrast to our observations for the drought treatment, the levels of chlorophyll and free proline exhibited a greater decline (Student’s *t*-test, *p* < 0.01 or *p* < 0.05) in the transgenic plants than in the WT plants after salt treatment (Figure 5B,D), but the levels of MDA had significantly increased (Student’s *t*-test, *p* < 0.05) (Figure 4). These results suggest that overexpression of the native *SlbZIP38* gene reduces salt stress tolerance.

### 3.6. Expression Analysis of the ABA Signaling Pathway Marker Genes under Drought and Salt Stress

**Figure 6:**
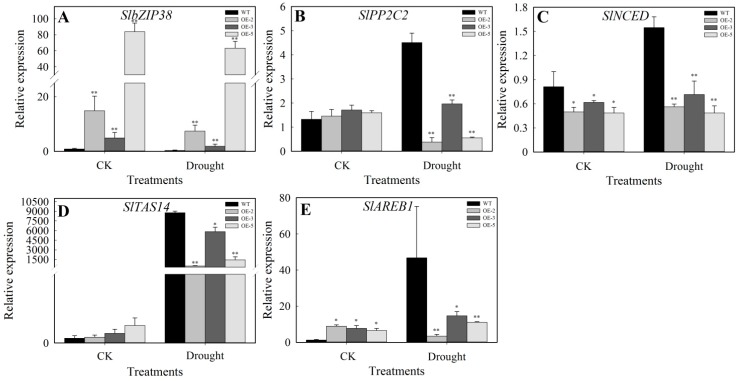
Relative mRNA transcript levels of *SlbZIP38* and ABA-responsive genes in WT and *SlbZIP38*-overexpressing lines under control and drought stress conditions. (**A**) *SlbZIP38*, (**B**) *SIPP2C2*, (**C**) *SlNCED*, (**D**) *SITAS14*, (**E**) *SlAREB1*. One-month-old WT and *SlbZIP38*-overexpressing plants were grown under drought conditions for seven days. The leaves were harvested and total RNA was isolated and subjected to RT-qPCR analysis. CK, samples from untreated plants serving as the control. Data represent the means ± SE (*n =* 3). Significantly different values from WT at * *p* < 0.05 and ** *p* < 0.01 level, respectively, as determined by Student’s *t*-test.

To confirm whether overexpression of *SlbZIP38* affected the ABA sensitivity (Figure 2E) of the transgenic plants, we thus analyzed the expression of *SlbZIP38* and four marker genes of ABA-responsive genes (*SlPP2C2*, *SlNCED*, *SlTAS14*, and *SlAREB1*) under drought and salt stress. The results showed that *SlbZIP38* expression is higher in transgenic than WT plants under normal and drought stress conditions (Figure 6A). In WT plants, the four ABA-responsive genes showed higher expression levels under dehydration treatment compared to the control, but in transgenic plants, the expression of these genes was decreased by this treatment (Figure 6B–E). No significant differences in *SlNCED* and *SlAREB1* expression were detected in the transgenic plants under normal and drought stress conditions (Figure 6C,E). The relative expression level of *SlTAS14* increased in the transgenic plants (Figure 6D), whereas the expression level of *SlPP2C2* was not significantly different in the OE-3 line, but was reduced in OE-2 and OE-5 plants (Figure 6B). In addition, *SlPP2C2*, *SlNCED*, and *SlAREB1* were expressed at lower levels in the transgenic plants than in the WT after drought treatment (Figure 6B–D). These results suggest that *SlbZIP38* regulates the expression of genes involved in ABA-related stress signaling pathways in tomatoes. These results support the finding that plants overexpressing *SlbZIP38* are less tolerant to drought than WT plants.

**Figure 7:**
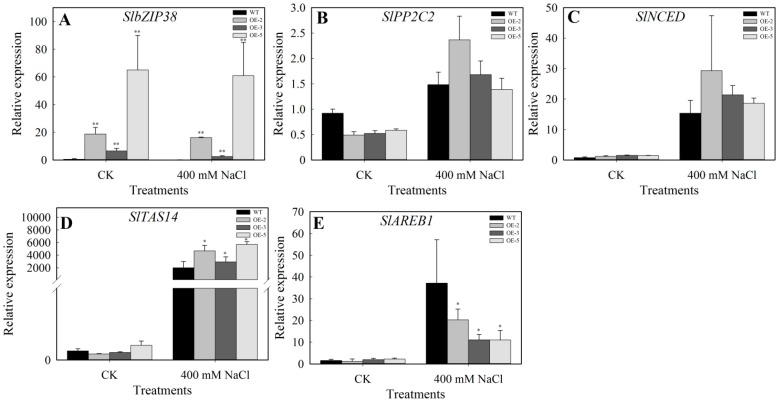
The relative mRNA transcript levels of *SlbZIP38* and ABA-responsive genes in WT and *SlbZIP38*-overexpressing lines in response to salt stress. (**A**) *SlbZIP38*, (**B**) *SIPP2C2*, (**C**) *SlNCED*, (**D**) *SITAS14*, (**E**) *SlAREB1*. One-month-old WT and *SlbZIP38*-overexpressing plants were treated with 400 mM NaCl for five days. The leaves were harvested and total RNA was isolated and subjected to RT-qPCR analysis. CK, samples from untreated plants serving as the control. Data represent the means ± SE (*n =* 3). Significantly different values from WT at * *p* < 0.05 and ** *p* < 0.01 level, respectively, as determined by Student’s *t*-test.

In line with the drought stress response, *SlbZIP38* expression was higher in transgenic plants than in the WT under normal and salt stress conditions (Figure 7A). The relative expression levels of *SlPP2C2*, *SlNCED*, *SlTAS14*, and *SlAREB1* were increased after salt treatment (Figure 7B–E). However, only *SlAREB1* displayed the same expression patterns under high salt and drought stress conditions in WT and transgenic plants (Figure 6E and Figure 7E). *SlPP2C2* and *SlNCED* gene were not significantly different between the transgenic plants and WT plants after salt treatment (Figure 7B,C), while the *SlTAS14* showed higher levels in transgenic plants than in WT plants after salt treatment (Figure 7D), indicating that the molecular mechanisms underlying *SlbZIP38* induction may differ depending on the type of stress or present. These results support the finding that plants overexpressing *SlbZIP38* are less tolerant to salt stress than WT plants.

## 4. Discussion

### 4.1. SlbZIP38 Is a Member of the Tomato bZIP Family

In the present study, we characterized a bZIP protein from tomatoes (SlbZIP38). Phylogenetic analysis showed that *SlbZIP38* formed a monophyletic group with *ZmbZIP72* (Figure 1A). Moreover, multiple alignment analysis showed that *SlbZIP38* has an amino acid sequence similar to that of *ZmbZIP72* (Figure 1B), which was previously shown to enhance drought and salt tolerance when heterologously expressed in *Arabidopsis*, suggesting that these genes may have similar functions [1]. In addition, the sequence comparison also showed that *SlbZIP38* contains the typically conserved DNA-binding domain (N-x7-R/K-x9), and a DOG1 structural domain of 79 amino acids among different plants (Figure 1B), suggesting that the encoded protein has conserved functions [37]. Extensive previous studies showed that numerous bZIP transcription factors could improve the tolerance of drought, cold and salt stress. The *ZmbZIP72* played important roles in abscisic acid (ABA) and stress signaling, which is similar to other bZIP proteins (i.e., *OsbZIP66* and *OsbZIP72*). In addition, some *SlbZIP* genes, *SlABZ1*, *SlAREB1*, and *LebZIP2* are also responsive to stress treatment [49,51,52]. Here, we found that *SlbZIP38* expression was significantly downregulated after ABA treatment (Figure 2E), indicating that this gene is negatively regulated by ABA stress. These data suggests that *SlbZIP38* might regulate the response to abiotic stressors through the ABA signaling pathway.

### 4.2. SlbZIP38-Overexpressing Plants Have a Reduced Tolerance to Drought and Salt Stress

Previous work has shown that *bZIP* genes are involved in the response to abiotic stressors such as drought, cold and high salinity [19,20]. In this study, we found that *SlbZIP38* had no effect or an inverse effect on the ability of a tomato plant to respond to high and low temperature, wilting and recovery, flooding, and salt (Figure 3A–D). In leaves, *SlbZIP38* expression was markedly and rapidly induced by exposure to a low temperature (Figure 3A), high temperature, wilting and recovery, and NaCl (Figure 3A,B,D), indicating that SlbZIP38 might play a role in regulating abiotic stress tolerance. In addition, we obtained 11 transgenic plants with significant levels of *SlbZIP38* (Figure 4A). Meanwhile, the drought and salt tolerance of the transgenic plants were significantly decreased compared to the WT (Figure 4B and Figure 5A). Moreover, there were no significant differences in chlorophyll, MDA and free proline content between the transgenic and WT plants before exposure to drought and salt stresses, but the MDA and free proline are markedly increased after drought at seven days and salt at five days (Figure 4C–E and Figure 5B–D). In addition, the chlorophyll content and free proline content of transgenic plants were significantly lower than that of the WT after drought and salt stress (Figure 4C,E and Figure 5B,D). Conversely, the higher MDA levels were detected in transgenic plants than in WT (Figure 4D and Figure 5C). These results are consistent with the previous results that the chlorophyll, MDA and free proline levels were used as a protective effect against environmental stressors [71,74,75,76,77]. Our findings suggest that *SlbZIP38* expression induces oxidative stress associated with drought and salt, thus making the *SlbZIP38*-overexpressing plants suffer more from oxidative damage than the WT plants.

### 4.3. SlbZIP38 Is a Negative Regulator of Drought and Salt Tolerance that Functions via the ABA Signaling Pathway

A number of bZIP genes (e.g., *AtbZIP1*, *OsbZIP23*, *OsbZIP66*, and *ZmbZIP72*) have been shown to play roles in the ABA signaling pathway [26,30,31,78,79]. In this study, we observed that *SlbZIP38*, a tomato bZIP family gene downregulated by Abscisic Acid (Figure 2D), and overexpression of *SlbZIP38* reduced drought and salt tolerance (Figure 4B and Figure 5A), indicating that *SlbZIP38* might be a negative regulator of drought and salt stress tolerance. These findings suggest that *SlbZIP38*’s response to drought and salt stress is regulated by the ABA signaling pathway. 

To understand the molecular mechanism between *SlbZIP38* and ABA signaling pathway well, we monitored the expression of four ABA-responsive genes (*SlPP2C2*, *SlNCED*, *SlTAS14*, and *SlAREB1*) before and after drought stress treatment. The four genes responded in various ways to different stressors and ABA treatment. *SlPP2C2* is an important ABA signaling molecule that showed both ABA-independent and ABA-dependent interactions with ABA receptor PYLs (Pyrabactin Riesistance 1-Llike). *SlPP2C2* is a putative ortholog of *AtPP2XA*, which is a negative regulator of the ABA signaling pathway in Arabidopsis [58,62]. *SlNCED* is the first rate-limiting enzyme in the ABA biosynthesis pathway [59,80,81]. *SlTAS14* encodes a dehydrin protein (a type 2 class of LEA protein) known to be induced by ABA and NaCl [61,82]. Furthermore, LEA genes play crucial roles in stabilizing labile enzymes and protecting membrane structures when plants are exposed to abiotic stress [60,83]. *SlAREB1* is a member of the AREB (abscisic acid-responsive element binding protein)/ABF (abscisic acid-responsive element binding factor) subfamily of bZIP transcription factors and is involved in abiotic stress-related ABA signaling [49,84]. In the present study, we analyzed the expression of ABA-related genes in the leaves of transgenic *SlbZIP38-*overexpression lines under drought and salt treatment and compared expression levels with those in the WT. The expression levels of *SlPP2C2*, *SlNCED*, *SlTAS14*, and *SlAREB1* in the transgenic plants were significantly decreased (Student’s *t*-test, *p* < 0.01) compared with those of WT plants when subjected to drought stress (Figure 6B–E), indicating that *SlbZIP38* is a negative regulator for ABA-dependent stress signal transduction. In addition, our results showed that *SlNCED* and *SlAREB1* transcript levels differed between the WT and transgenic plants both before and after exposure to drought stress (Figure 6C,E), suggesting that the expression levels of *SlPP2C2* and *SlTAS14* were readily induced by drought stress in the transgenic plants. Conversely, the relative expression levels of *SlPP2C2*, *SlNCED*, *SlTAS14*, and *SlAREB1* were also increased after salt treatment (Figure 7B–E). The *SlAREB1*, which is considered a marker of the ABA signaling pathway [49,84], had similar expression patterns under drought and salt stress in WT and transgenic plants (Figure 6E and Figure 7E), implying that *SlAREB1* might activate *SlbZIP38* expression during exposure to drought and salt stress, and that *SlbZIP38* acts by modulating the ABA signaling pathway. For the SlTAS14, which encodes for a dehydrin (a type 2 class of LEA protein) whose expression in tomatoes is known to be induced by ABA, mannitol and NaCl [50]. However, the expression of *SlTAS14* exhibited a greater increase under drought stress than salt stress; while expression of this gene was greater than that of the WT under salt stress, it was less than that of the WT under drought (Figure 6D and Figure 7D). These results showed that the molecular mechanism underlying *SlbZIP38* expression was different under drought and salt stress conditions. Over-expression of *SlbZIP38* only decreased the *SlAREB1* expression but induced the *SlTAS14* compared with WT during the salt stress, indicating that *SlTAS14* may not only be involved in the ABA signal pathway but also be involved in other pathways during the salt stress. Taken together, these data suggest that *SlbZIP38* is a negative regulator of drought and salt stress. Therefore, the molecular mechanisms of their action might be interpreted by further studies in the future works.

## 5. Conclusions

In this study, we cloned and characterized *SlbZIP38* from tomatoes. Overexpression of *SlbZIP38* resulted in decreased tolerance to drought and salt stress. Our results indicate that *SlbZIP38* suppresses the plant’s drought stress response through inducing of the downstream pathway of ABA signaling. The mechanism by which *SlbZIP38* functions in the plant’s response to abiotic stress remains to be further investigated. 

## Figures and Tables

**Table 1:** Information of primers for this manuscript.

Gene Name	Primer Sequences (5′-3′)	
*SlbZIP38*	CCATGCAAGCTTTCAAAGAAGCAGCTGT	GAGATGAATACGACGTACTAGAGTTGG
*SlbZIP38-*Q	GAGGTGTTTCATGTGGTTAGGTGGAT	CGGCTTGCTGAGAAGACTGTTGC
*SlTAS14-*Q	AGAAGGTGGGAGGAGAAAGAAG	ATGGAGATGAAAACAAAGGTGTT
*SlNCED-*Q	CCGGTGGTTTACGACAAGAA	TCCAGAGGTGGAAACAGAAAC
*SlPP2C-*Q	CAGTGATGGATTATGGGACGTGGTA	CCTAGCCAAGGCTAATTTCGTCAA
*SlAREB1-*Q	CAGGTGAGGGTGGAAGTGGTGGTGG	TGTTTGATTCTCCTCAGCATTCCAT
*SlELF-α*	ACCTTTGCTGAATACCCTCCATTG	CACACTTCACTTCCCCTTCTTCTG
